# Identification of the Biosynthetic Pathway of Glycine Betaine That Is Responsible for Salinity Tolerance in Halophilic *Thioalkalivibrio versutus* D301

**DOI:** 10.3389/fmicb.2022.875843

**Published:** 2022-04-18

**Authors:** Mengshuang Liu, Hui Liu, Fangtong Mei, Niping Yang, Dahe Zhao, Guomin Ai, Hua Xiang, Yanning Zheng

**Affiliations:** ^1^State Key Laboratory of Microbial Resources, Institute of Microbiology, Chinese Academy of Sciences, Beijing, China; ^2^College of Life Science, University of Chinese Academy of Sciences, Beijing, China; ^3^CAS Key Laboratory of Bio-based Materials, Qingdao Institute of Bioenergy and Bioprocess Technology, Chinese Academy of Sciences, Qingdao, China; ^4^College of Environment, Hohai University, Nanjing, China; ^5^School of Life Sciences, Hebei University, Baoding, China

**Keywords:** glycine betaine, biosynthetic pathway, *Thioalkalivibrio versutus*, glycine *N*-methyltransferase, sarcosine dimethylglycine *N*-methyltransferase

## Abstract

*Thioalkalivibrio versutus* D301 has been widely used in the biodesulfurization process, as it is capable of oxidizing hydrogen sulfide to elemental sulfur under strongly halo-alkaline conditions. Glycine betaine contributes to the increased tolerance to extreme environments in some of *Thioalkalivibrio* species. However, the biosynthetic pathway of glycine betaine in *Thioalkalivibrio* remained unknown. Here, we found that genes associated with nitrogen metabolism of *T. versutus* D301 were significantly upregulated under high-salt conditions, causing the enhanced production of glycine betaine that functions as a main compatible solute in response to the salinity stress. Glycine betaine was synthesized by glycine methylation pathway in *T. versutus* D301, with glycine *N*-methyltransferase (GMT) and sarcosine dimethylglycine *N*-methyltransferase (SDMT) as key enzymes in this pathway. Moreover, substrate specificities of GMT and SDMT were quite different from the well characterized enzymes for glycine methylation in halophilic *Halorhodospira halochloris*. Our results illustrate the glycine betaine biosynthetic pathway in the genus of *Thioalkalivibrio* for the first time, providing us with a better understanding of the biosynthesis of glycine betaine in haloalkaliphilic *Thioalkalivibrio*.

## Introduction

Microbes face many challenges in halo-alkaline environments, which impose high extracellular osmotic pressures on microbial cells. To avoid the outflow of intracellular water and maintain the functions of biomacromolecules, microbes mainly adopt “salt-in” and “compatible solute” strategies to balance the intra- and extracellular osmotic pressures (Gunde-Cimerman et al., 2018). The “salt-in” strategy used by many halophilic archaea and a few halophilic bacteria is to increase the intracellular osmotic pressure by accumulating high concentrations of inorganic salts (mainly KCl) (Christian and Waltho, 1962; Ginzburg et al., 1970; Eisenberg and Wachtel, 1987; Gunde-Cimerman et al., 2018). Microorganisms that use salt-in strategy usually have an acidic proteome to adapt to the intracellular high-salt content, which is necessary for acidic proteins to maintain their structural stabilities and functional activities (Reistad, 1970; Lanyi, 1974; Dennis and Shimmin, 1997). Therefore, microorganisms utilizing “salt-in” strategy are highly dependent on high-salt environments and generally unable to survive under low-salt conditions. Microorganisms that use “compatible solute” strategy can synthesize or import small organic molecules called compatible solutes to maintain the osmotic balance of cells (Kempf and Bremer, 1998). The compatible solutes mainly include sugars, polyols, amino acids, and their derivatives (Galinski and Truper, 1994; Roberts, 2005). The accumulation of compatible solutes will increase the intracellular osmolarity without interfering with the normal cellular activities (Brown, 1976). Given microorganisms using compatible solutes can better adapt to environmental fluctuations than those using “salt-in” strategy, “compatible solute” strategy is more widely adopted by microbes inhabiting halo-alkaline environments.

Haloalkaliphilic *Thioalkalivibrio* species are a class of obligate chemoautotrophs using reduced sulfur compound as energy source and carbon dioxide (CO_2_) as carbon source (Sorokin et al., 2001). They live in environments of different pHs and salinities, ranging from 7.5 to 10.5 and 0.3 to 4.0 M Na^+^, respectively (Sorokin et al., 2001). Members of *Thioalkalivibrio* genus mainly employ “compatible solute” strategy to balance the intra- and extracellular osmotic pressures and then survive in high-salt environments (Banciu et al., 2004a,b, 2005). N-containing glycine betaine was detected in *Thioalkalivibrio halophilus* when grown in 4 M NaCl and 4 M soda media, with osmotic pressures of 9.3 and 5 osm/kg, respectively (Banciu et al., 2004a). The extracellular osmotic pressure but not just the concentration of Na^+^ determines the biosynthesis of glycine betaine, given that a higher osmotic pressure contributes to a higher level of glycine betaine. Besides, a osmolarity-dependent production of glycine betaine was also observed in *Thioalkalivibrio versutus* ALJ 15 (Banciu et al., 2005). In addition, glycine betaine confers resistance to the low-temperature pressure in two moderately halophilic *Thioalkalivibrio* strains (Ahn et al., 2021). Though glycine betaine is of great importance for *Thioalkalivibrio* to keep them alive in extreme environments, the biosynthetic pathway of glycine betaine is still unclear in *Thioalkalivibrio* species.

**FIGURE 1:**
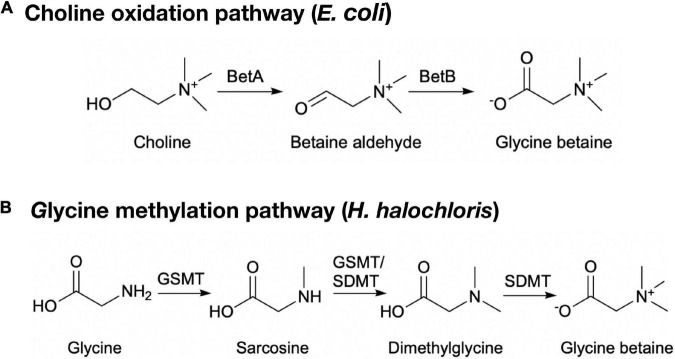
Glycine betaine biosynthetic pathways in microorganisms. **(A)** The choline oxidation pathway in *E. coli*. BetA, choline dehydrogenase; BetB, betaine-aldehyde dehydrogenase. **(B)** The glycine methylation pathway in *H. halochloris.* GSMT, glycine sarcosine *N*-methyltransferase; SDMT, sarcosine dimethylglycine *N*-methyltransferase.

The choline oxidation pathway and glycine methylation pathway are two pathways that have already been known for the biosynthesis of glycine betaine (Figure 1). In the choline oxidation pathway, choline is converted to glycine betaine by choline dehydrogenase and betaine-aldehyde dehydrogenase, with betaine-aldehyde as the intermediate (Landfald and Strom, 1986; Boyd et al., 1991). In addition, choline oxidase alone is also able to convert choline to glycine betaine (Fan et al., 2004). In the glycine methylation pathway, S-adenosyl-L-methionine (SAM)-dependent *N*-methyltransferases catalyze the conversion of glycine to glycine betaine by the addition of three methyl groups to its amino group, with sarcosine and dimethylglycine as the intermediates. The substrate specificities of SAM-dependent *N*-methyltransferases that catalyze the conversion of glycine to glycine betaine vary from organism to organism. A single glycine sarcosine dimethylglycine *N*-methyltransferase (TpGSDMT) can complete the whole conversion process in *Thalassiosira pseudonana* (Kageyama et al., 2018). More commonly, two enzymes are involved in the three methylation reactions in halophiles, such as glycine sarcosine *N*-methyltransferase (GSMT)/sarcosine dimethylglycine *N*-methyltransferase (SDMT) in *Halorhodospira halochloris* (formerly *Ectothiorhodospira halochloris*) and *Methanohalophilus portucalensis* (Nyyssola et al., 2000, 2001; Lai and Lai, 2011), and glycine sarcosine *N*-methyltransferase (GSMT)/dimethylglycine *N*-methyltransferase (DMT) in *Aphanothece halophytica* and *Synechococcus sp*. (Waditee et al., 2003; Lu et al., 2006).

*Thioalkalivibrio versutus* D301, a strain widely used in biodesulfurization industry, contains a 2,969,361-bp circular chromosome (Mu et al., 2016, 2021). The genes coding for choline oxidation pathway are absent in the genome of *T. versutus* D301, but a set of chromosomal genes (*TVD_RS00875* and *TVD_RS00880*) homologous to the *GSMT* and *SDMT* genes from *H. halochloris* are available. Though *Thioalkalivibrio* has a close phylogenetic relationship to *Halorhodospira*, it is still unclear whether SAM-dependent *N*-methyltransferases from *Thioalkalivibrio* species also catalyze the conversion of glycine to glycine betaine (Nyyssola et al., 2001; Sorokin et al., 2001). So far, no study has been carried out to characterize the glycine methylation pathway of *Thioalkalivibrio* species. Here, we show that N-containing glycine betaine is a main compatible solute in *Thioalkalivibrio versutus*. We also show that the glycine *N*-methyltransferase (TvGMT) and sarcosine dimethylglycine *N*-methyltransferase (TvSDMT) are responsible for the conversion of glycine to glycine betaine by adding three methyl groups to the amino group of glycine.

## Materials and Methods

### Bacterial Strains and Growth Conditions

*Thioalkalivibrio versutus* D301 was grown aerobically on a slightly modified TD medium supplemented with 10 g/L NaHCO_3_ (Low-salt medium, 0.4 M Na^+^) or 10 g/L NaHCO_3_ plus 152 g/L NaCl (High-salt medium, 3.0 M Na^+^) at 30°C and 200 rpm (Mu et al., 2016, 2017). The pH of the modified TD media was adjusted to 9.5 with 2 M hydrochloric acid. *E. coli* BL21(DE3) grown in Luria-Bertani (LB) medium was used to overexpress the target enzymes of interest. Cultures of *E. coli* growing at 37° and 200 rpm were switched to incubation at 30° and 160 rpm for overexpression of proteins. When appropriate, *E. coli* cultures were supplemented with kanamycin at 50 μg/mL.

### Transcriptome Sequencing and Functional Enrichment Analysis of Differentially Expressed Genes

*Thioalkalivibrio versutus* cultures grown under low-salt and high-salt conditions were collected at the late exponential growth phase for transcriptome sequencing, which was carried out by Majorbio Bio-Pharm Technology Co., Ltd. (Shanghai, China). The transcriptome sequencing data were deposited in NCBI^1^ with BioProjects accession number PRJNA812740. After the quality control of raw reads was completed, clean reads were obtained and then mapped to the reference genome of *T. versutus* D301 (CP011367) using Bowtie 2 (version 2.3.5) (Mu et al., 2016). The fragments per kilobase of transcript per million mapped reads (FPKM) calculated by RSEM (version 1.3.1) was used to represent the expression level of genes under low-salt and high-salt conditions. Differentially expressed genes (DEGS) of *T. versutus* D301 were identified using DESeq2 (version 1.24.0) based on the value of | log2 fold change| > 2 and an adjusted *P* < 0.05. The Gene Ontology (GO) functional enrichment analysis of DEGS were performed by Goatools.

### LC/MS Analysis of Intracellular Metabolites

For the LC/MS analysis of intracellular metabolites, 50 mL cultures of *T. versutus* D301 grown under low-salt and high-salt conditions, respectively, were harvested at the late exponential growth phase. Firstly, the cultures were centrifuged at 10,000 rpm for 20 min to collect the pellets, then the cell pellets were washed twice with 0.4 or 3 M NaCl solution, and finally 1 mL ultrapure water was used to resuspend the pellets. The cell lysates were prepared by treating the obtained cell suspensions with four freeze-thaw cycles: froze at −80°C for 15 min and thawed at 65°C for 2 min. Cell lysates were centrifuged at 12,000 rpm for 30 min to collect the supernatants, which were further mixed with acetonitrile in a ratio of 3:7 (v/v). After the mixtures were centrifuged at 12,000 rpm for 10 min, the supernatants filtered with a 0.22 μm nylon filter membrane were analyzed with LC/MS (ESI). Chromatography was performed with an Agilent 1260/6460 LC/Triple Quad MS system, using a TSKgel NH_2_-100 column (2.0 × 150 mm, 3 μm; TOSOH, Tokyo, Japan) with guard column (2.0 × 10 mm, 3 μm). Mobile phase A was 10 mM ammonium formate supplemented with 0.07% (v/v) formic acid, while mobile phase B was pure acetonitrile. The following method was used with a flow rate of 0.25 ml/min: 85% mobile phase B for 2 min; decrease of 1.5% mobile phase B/min to 55% mobile phase B; holding at 55% mobile phase B for 5 min; increase of 15% mobile phase B/min to 85% mobile phase B; holding at 85% mobile phase B for 15 min. Mass spectra were acquired in positive ionization mode, with a fragmentor of 80 V and a scan range of 70.0–1000.0 m/z. Data analysis was performed using Agilent MassHunter Qualitative Analysis B.04.00 Workstation Software.

### Quantification of Glycine Betaine by HPLC

Samples used for quantification of intracellular glycine betaine were prepared using the same method as what mentioned in “LC/MS analysis of intracellular metabolites”. Agilent 1260 Infinity II system equipped with a Inertsil NH_2_ column (4.6 × 250 mm, 5 μm, GL Sciences, Tokyo, Japan) was used to measure the glycine betaine. Acetonitrile/ultrapure water (70:30, v/v) was used as the mobile phase at a flow rate of 1 mL/min. The detection wavelength of 196 nm was used to measure the glycine betaine. Total protein concentrations were determined using the Bradford method.

### Protein Expression and Purification

The *TVD_RS00875* and *TVD_RS00880* genes, coding for the putative glycine methylation pathway, were inserted into *Bam*HI-digested pET-28a(+), respectively, using the T5 exonuclease-dependent assembly system (Xia et al., 2019). The obtained *E. coli* strains grown with 50 μg/mL kanamycin were used to overexpress TVD_RS00875 (TvGMT) and TVD_RS00880 (TvSDMT) after addition of 0.05 mM IPTG. Cell extracts were prepared by high pressure homogenization in buffer A (20 mM Tris–HCl, 300 mM NaCl, 20 mM imidazole, 2 mM DTT, pH 7.5). His-tagged proteins were purified by an affinity column packed with Ni Sepharose (Cytiva, Uppsala, Sweden). After the pretreated sample loaded onto the affinity column was washed with 10 column volumes of buffer A and then 5 column volumes of 5% buffer B (20 mM Tris–HCl, 300 mM NaCl, 44 mM imidazole, 2 mM DTT, pH 7.5), TvGMT or TvSDMT was eluted using 30% buffer B (20 mM Tris–HCl, 300 mM NaCl, 164 mM imidazole, 2 mM DTT, pH 7.5). Protein concentrations were determined by Bradford assay using BSA as standard. The purities of TvGMT and TvSDMT were examined based on SDS-PAGE analysis with Coomassie staining.

### Methyltransferase Assay

Methyltransferase activity was measured using SAM as the methyl donor and glycine, sarcosine, or dimethylglycine as methyl receptor. The reaction mixture contained 100 mM Tris–HCl (pH 7.5), 12.5 μmol MgCl_2_, 0.5 mM DTT, 10 mM SAM, 250 mM glycine/30 mM sarcosine (TvGMT) or 60 mM sarcosine/50 mM dimethylglycine (TvSDMT) (Nyyssola et al., 2001). Reactions that initiated by the addition of TvGSMT or TvSDMT were conducted at 37°C for 30 min and then quenched by heating with boiling water for 10 min. The supernatants obtained by centrifugation were collected for HPLC analysis of products. The standard curves used for quantification of products were made with the commercially available sarcosine, dimethylglycine, and glycine betaine, respectively.

## Results

### Genes Involved in Nitrogen Metabolism Are Significantly Upregulated Under High-Salt Conditions

**FIGURE 2:**
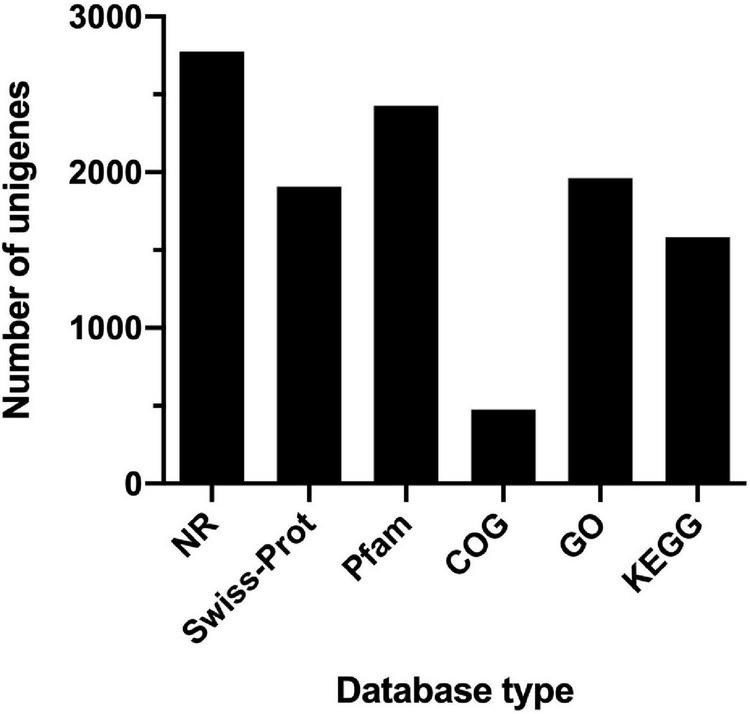
The statistic histogram of basic functional annotation of genes in *T. versutus* D301. The horizontal axis represents different database used for annotation. The vertical axis represents the number of genes annotated by different database. The total number of coding genes is 2,788 in *T. versutus* D301.

**FIGURE 3:**
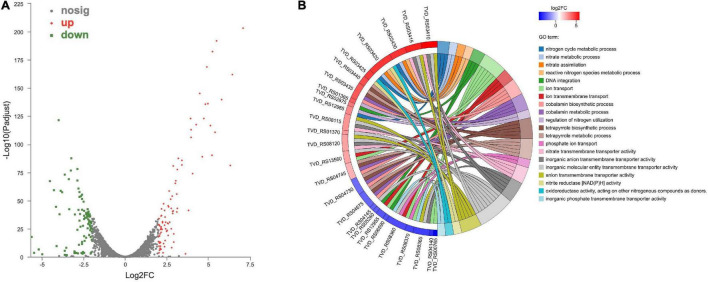
Transcriptomic analysis of differentially expressed genes (DEGs) between high-salt and low-salt conditions. **(A)** Volcano plot of the DEGs. Red dots indicate significantly upregulated genes, while green squares indicate significantly downregulated genes. The |log2FC| > 2 and an adjusted *P* < 0.05 was used as threshold values. **(B)** The top twenty GO enrichment terms from DEGs.

The comparative transcriptomic analysis was carried out to examine the response of *T. versutus* to high-salt stress. *T. versutus* cultures were firstly grown under low-salt and high-salt conditions, and cells were then harvested at the late exponential phase. After the cDNA libraries were constructed, they were sequenced using the Illumina HiSeq platforms. Around 4 Gb clean data for each sample was obtained after the quality control of raw data. The information of transcriptome sequencing data was shown in Table 1. Clean reads of each sample were mapped to the reference genome of *T. versutus* D301 (CP011367), with alignment rates ranging from 98.77 to 99.25%. The functional information of *T. versutus* D301 genome was obtained through the annotation of non-redundant protein (NR), Swiss-Prot, Pfam, COG, GO and KEGG Database (Figure 2). The FPKM was used to measure the expression levels of genes or transcripts. A total of 184 differentially expressed genes (DEGs) were obtained between *T. versutus* cultures grown under high-salt and low-salt conditions, with 90 genes significantly upregulated and 94 genes significantly downregulated (Figure 3A).

**TABLE 1:** Summary of transcriptome sequencing^#^.

Sample	Raw reads	Clean reads	Clean bases	Error rate (%)	Q20 (%)	Q30 (%)
D-1	32145804	31895494	4.50 Gb	0.0228	98.93	96.46
D-2	31633772	31464864	4.34 Gb	0.0226	99	96.63
G-1	31211978	31028832	4.25 Gb	0.0228	98.93	96.45
G-2	27721942	27565292	3.72 Gb	0.0226	99	96.65

*^#^D-1, D-2: Two duplicate samples cultured under low-salt condition (0.4 M Na^+^); G-1, G-2: Two duplicate samples cultured under high-salt condition (3.0 M Na^+^).*

To further understand the physiological response of *T. versutus* to high-salt stress, we performed GO enrichment analysis of DEGs (Figure 3B). The nitrate transmembrane transporter activity, nitrogen cycle metabolic process, nitrate metabolic process, nitrate assimilation, reactive nitrogen species metabolic process and nitrite reductase [NAD(P)H] activity were among the top 20 GO terms. As shown in Table 2, as much as two-thirds of the top 20 significantly upregulated genes were involved in nitrogen metabolism or related regulation process.

**TABLE 2:** The significantly upregulated genes related to nitrogen metabolism^#^.

Protein name	Gene ID	FPKM		FC	Log_2_FC	Function
		High salt	Low salt			
NA	TVD_RS03400	134.21	3.615	42.323	5.403	Nitrate regulatory protein
NrtA	TVD_RS03405	30650.935	251.76	136.09	7.088	Nitrate uptake
	TVD_RS08095	907.85	32.615	31.302	4.968	
	TVD_RS08110	8216.5	1522.26	6.031	2.592	
NrtB	TVD_RS03410	15043.425	306.04	55.296	5.789	
	TVD_RS08100	567.245	17.045	38.056	5.250	
	TVD_RS08115	7561.32	1158.695	7.383	2.884	
NrtC	TVD_RS03415	32590.38	1054.955	34.907	5.125	
	TVD_RS08090	809.25	10.55	86.569	6.436	
	TVD_RS08120	7568.27	1412.43	6.069	2.602	
NasA	TVD_RS03430	4498.09	179.755	27.957	4.805	NO_3_^–^ → NO_2_^–^
NirB	TVD_RS03420	9293	391.055	26.506	4.728	NO_2_^–^ + NADH + H^+^ → NH_3_ + NAD^+^ + H_2_O
NirD	TVD_RS03425	3084.07	147.525	24.842	4.635	
GlnA	TVD_RS13790	11191.13	1517.44	8.316	3.056	L-Glutamate + NH_3_ + ATP → L-Glutamine + ADP + Pi
GlnK	TVD_RS01365	7960.275	607.495	15.556	3.959	P-II family nitrogen regulator
GlnG	TVD_RS12985	452.58	57.905	8.743	3.128	Nitrogen regulation protein NR(I)
GlnL	TVD_RS12990	120.695	18.755	7.318	2.871	PAS domain-containing sensor histidine kinase
Amt	TVD_RS01370	22694.515	3576.315	7.144	2.837	Ammonium transporter
NifA	TVD_RS08105	57.84	0.825	79.789	6.318	Sigma-54-dependent Fis family transcriptional regulator
CynS	TVD_RS08125	15042.885	3917.965	4.493	2.168	Cyanate + HCO_3_^–^ + H^+^ → NH_3_ + CO_2_

*^#^NA, Not annotated.*

Glycine betaine and ectoine/hydroxyectoine are N-containing compatible solutes commonly found in halophiles. Two candidate genes *TVD_RS00875* and *TVD_RS00880* probably encoding the enzymes for glycine methylation pathway were found in the genome of *T. versutus*, with the putative genes coding for choline oxidation pathway and ectoine/hydroxyectoine biosynthetic pathway absent. However, no significant difference was observed in transcriptional levels of *TVD_RS00875* and *TVD_RS00880* between high-salt and low-salt conditions. The transcriptional levels 6∼14 times higher than the *rpoN* gene encoding sigma 54.

### N-Containing Glycine Betaine Is a Main Compatible Solute in *Thioalkalivibrio versutus* D301

**FIGURE 4:**
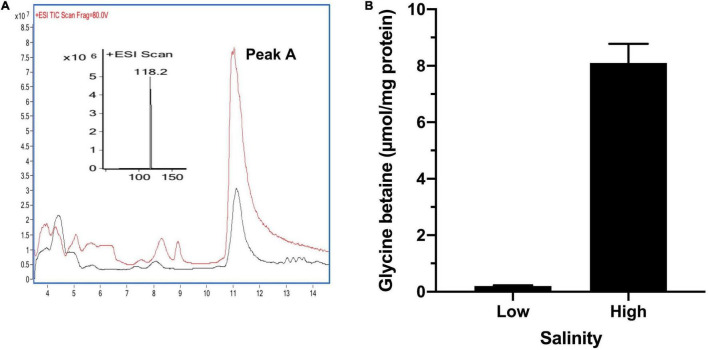
Qualitative and quantitative analysis of glycine betaine in *T. versutus* D301 grown under high-salt (3.0 M) and low-salt (0.4 M) conditions, respectively. **(A)** The red and black traces are the LC-MS data for monitoring protonated glycine betaine (m/z = 118) under high-salt and low-salt conditions, respectively. **(B)** Quantitative analysis of glycine betaine production by HPLC. Data are the average of three biological replicates and the error bars represent the s.d.

To confirm that the glycine betaine was responsible for the high-salt tolerance in *T. versutus* D301, we measured the glycine betaine in *T. versutus* D301 grown in high-salt (3.0 M Na^+^) and low-salt (0.4 M Na^+^) media, respectively. Intracellular metabolites of *T. versutus* D301 were firstly analyzed qualitatively by LC/MS. *T. versutus* D301 grown under high-salt conditions produced a compound with an m/z ratio of 118 and a liquid chromatography retention time corresponding to glycine betaine (Figure 4A). The glycine betaine contents were then determined quantitatively by HPLC. *T. versutus* D301 produced much more glycine betaine under high-salt conditions (8.1 μmol/mg total protein) compared to the production under low-salt conditions (0.2 μmol/mg total protein), demonstrating that glycine betaine is indeed a compatible solute to resist the high-salt stress in *T. versutus* D301 (Figure 4B). However, in this range of salt concentrations, the contents of glycine betaine increases were not linearly with an increase in salt concentration.

### Glycine Betaine Is Synthesized *via* Glycine Methylation Pathway in *Thioalkalivibrio versutus* D301

**FIGURE 5:**
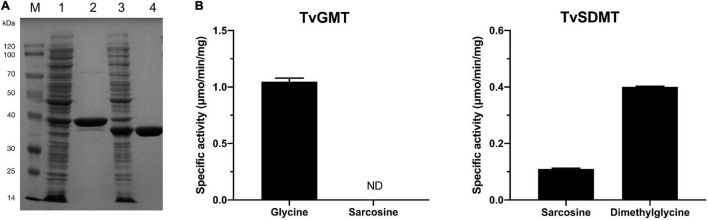
Glycine *N*-methyltransferase (TvGMT) and sarcosine dimethylglycine *N*-methyltransferase (TvSDMT) overexpressed and purified from *E. coli* convert glycine to glycine betaine by sequentially transferring three methyl groups to glycine. **(A)** SDS-PAGE analysis of TvGMT and TvSDMT. Lane M, protein marker; Lane 1, crude extract of TvGMT; Lane 2, purified TvGMT; Lane 3, crude extract of TvSDMT; Lane 4, purified TvSDMT. **(B)** Specific activities of TvGMT and TvSDMT using glycine, sarcosine, and dimethylglycine as substrates, respectively. Data are the average of three biological replicates and the error bars represent the s.d.

To determine if glycine methylation pathway is used by *T. versutus* D301 for the biosynthesis of glycine betaine, His-tagged versions of the TVD_RS00875 and TVD_RS00880 were overexpressed and purified from *E. coli* BL21(DE3) (Figure 5A). The calculated molecular masses based on the amino acid sequences of the TVD_RS00875 and TVD_RS00880 are both 32 kDa. However, the molecular mass of TVD_RS00875 estimated from the SDS-PAGE gel was slightly higher than its calculated molecular mass. A similar phenomenon was also observed for HhGSMT from *H. halochloris* (Nyyssola et al., 2001). The molecular masses of HhGSMT estimated from the SDS-PAGE and analytical gel filtration were 42 kDa and 40 kDa, both of which were far higher than the calculated molecular mass of 31 kDa.

Methyltransferase assays were conducted according to a published method with minor modifications (Figure 5B; Nyyssola et al., 2001). Given that the reaction product S-adenosylhomocysteine (AdoHcy) is a strong competitive inhibitor of many methyltransferases (Heady and Kerr, 1973; Upmeier et al., 1988; Nyyssola et al., 2001), as high as 10 mM SAM was used as the methyl donor in the reaction system. The purified TVD_RS00875 converted glycine to sarcosine with a relatively high specific activity (1.0 U/mg protein). The substrate specificity of TVD_RS00875 is different from HhGSMT, which transfers methyl group to both glycine and sarcosine (Nyyssola et al., 2001). Therefore, the TVD_RS00875 was designated as TvGMT. The purified TVD_RS00880, designated as TvSDMT, exhibited activities on both sarcosine and dimethylglycine, showing a higher specific activity toward dimethylglycine. The sequential action of TvGMT and TvSDMT resulted in the biosynthesis of glycine betaine from glycine in a process of three-step methylation.

## Discussion

The comparative transcriptomic analysis showed that genes involved in nitrogen metabolism were significantly upregulated under high-salt conditions. The upregulated genes for nitrate ABC transporters (NrtABC) (Frias et al., 1997), with log_2_FC ranging from 2.6 to 7.1, could result in the transport of more extracellular nitrate into the cytosol of *T. versutus*, where nitrate is sequentially reduced to ammonia by nitrate reductase (NasA, TVD_RS03430) and nitrite reductase (NirBD, TVD_RS03425 and TVD_RS03420), respectively, with nitrite as the intermediate (Harborne et al., 1992; Ogawa et al., 1995). The *nasA* and *nirBD* genes were all significantly upregulated (log_2_FC > 4) in response to high-salt stress. Besides the nitrate reduction, ammonia transport and assimilation genes such as *amt* (*TVD_RS01370*) and *glnA* (*TVD_RS13790*), whose gene products ammonia transporter (Amt) and glutamine synthetase (GlnA) are responsible for the transport of extracellular ammonia into cells and for the conversion of ammonia and glutamate to glutamine, respectively, were also significantly upregulated. Besides the genes directly involved in nitrogen metabolism, genes associated with their regulation were also upregulated, such as two-component system GlnLG (*TVD_RS12990* and *TVD_RS12985*) that responds to the nitrogen limitation and then activate the expression of *glnA* (Reitzer, 2003). Therefore, pathways associated with nitrogen metabolism could play a key role in resisting the high-salt stress.

Sufficient supply of nitrogen is required to guarantee the biosynthesis of N-containing compatible solutes. Marine bacterium *Dinoroseobacter shibae*, which normally uses both N-containing glutamate and N-free α-glucosylglycerate/α-glucosylglycerol as compatible solutes, prefers to synthesize α-glucosylglycerate when nitrogen is limiting (Kleist et al., 2017). Besides *D. shibae*, halophilic bacterium *H. halochloris* produces more trehalose and less glycine betaine to maintain the intra- and extracellular osmotic balance under nitrogen-limited conditions (Galinski and Herzog, 1990). The obvious upregulation of genes associated with nitrogen metabolism suggests that a variety of nitrogen sources are mobilized for use to guarantee the biosynthesis of N-containing compatible solutes. Given that N-containing glycine betaine is a compatible solute commonly used in *Thioalkalivibrio* species (Banciu et al., 2004a,^2005^; Ahn et al., 2021), the rapid mobilization of biologically available nitrogen is probably used for the biosynthesis of glycine betaine.

Based on the genomic analysis of *T. versutus* D301, *TVD_RS00875* and *TVD_RS00880* gene products that homologous to the GSMT and SDMT from *H. halochloris* were considered to be responsible for the biosynthesis of glycine betaine by the three-step methylation of glycine. No genes involved in choline oxidation pathway were found in the genome of *T. versutus* D301. However, transcriptomic analysis showed that the expression levels of both *TVD_RS00875* and *TVD_RS00880* under high-salt conditions did not increase compared to that under low-salt conditions. It demonstrates the biosynthesis of glycine betaine are probably regulated by posttranslational modification, and nitrogen metabolism related genes are regulated at transcriptional level. In addition to *de novo* biosynthesis of glycine betaine, *T. versutus* is also able to transport glycine betaine across membranes by glycine betaine/proline ABC transporters (TVD_RS10550, TVD_RS10555, and TVD_RS10560) when glycine betaine is available in environment (Ko and Smith, 1999). When glycine betaine is unavailable in extreme environment, *T. versutus* will synthesize compatible solutes to overcome the challenge of environmental osmolarity.

Different from *T. versutus* D301, which had an about 40-fold increase of glycine betaine content in response to high-salt stress as shown by quantification, *T. versutus* ALJ 15 grown in medium supplemented with a high concentration of sodium carbonate/sodium bicarbonate (4 M Na^+^, 0.1 M NaCl, and 3.9 M Na_2_CO_3_/NaHCO_3_) only produced six-fold more glycine betaine than in low-salt medium (0.6 M Na^+^, 0.1 M NaCl, and 0.5 M Na_2_CO_3_/NaHCO_3_) (Banciu et al., 2005). As measured by Banciu et al., the osmotic pressure of 4 M NaCl was almost two times higher than that of 4 M Na_2_CO_3_/NaHCO_3_ (Banciu et al., 2004a). Based on this measurement, the osmotic pressure of 2.6 M NaCl is slightly higher than that of 3.4 M Na_2_CO_3_/NaHCO_3_. Given that glycine betaine in *T. halophilus* grown with 4 M NaCl and 4 M soda medium accounted for 19.8% (w/w) and 12.4% (w/w) of biomass, respectively, such a small difference in osmolarity will not make such a large difference in the content of glycine betaine (Banciu et al., 2004a). It is worth noting that *T. versutus* ALJ 15 also produced a certain amount of sucrose under high-salt conditions (4 M Na^+^), accounting for 1.7% of the cell dry weight (Banciu et al., 2005). The N-free sucrose may partly contribute to the salinity tolerance of *T. versutus* ALJ 15. However, no differential expression of the putative sucrose-phosphate synthase gene (*TVD_RS01115*), which is responsible for the biosynthesis of sucrose in *T. versutus* D301, was observed between low-salt and high-salt conditions. Moreover, no sucrose could be detected in *T. versutus* D301 cells grown under low-salt and high-salt conditions, respectively. Therefore, different from the situation in *T. versutus* ALJ 15, glycine betaine probably plays a major role in resisting the high-salt stress in *T. versutus* D301 (Banciu et al., 2004a,^2005^).

This study shows that glycine methylation pathway is used by *T. versutus* D301 for the biosynthesis of glycine betaine, in which TvGMT and TvSDMT sequentially catalyze the three-step methylation of glycine. It is worth noting that the specific activity of TvGMT was over six-fold higher than that of HhGSMT (Nyyssola et al., 2001). Therefore, *TvGMT* can be used as a promising gene for the heterologous synthesis of glycine betaine in transgenic plants, which would be more tolerant to halo-alkaline environments (Chen and Murata, 2002). TvGMT and TvSDMT have about 77 and 59% sequence identities to HhGSMT and HhSDMT from *H. halochloris*, which were already known to participate in biosynthesis of glycine betaine *via* glycine methylation pathway (Nyyssola et al., 2001). In addition, the gene clusters encoding glycine methylation pathway were also present in the genomes of *Thioalkalivibrio sp*. and *T. sulfidophilus* (Muyzer et al., 2011a,b), suggesting that biosynthesis of glycine betaine *via* glycine methylation pathway is a general mechanism employed by *Thioalkalivibrio* species. However, the substrate specificity of TvGMT was quite different from HhGSMT. It suggests enzymes for glycine methylation pathway have evolved for different bacteria to adapt to changing environments. Characterization of these enzymes will contribute to a better understanding of the environmental adaptation mechanism of *Thioalkalivibrio*. The conversion of glycine to glycine betaine is energy intensive because of the requirement for SAM, which is involved in methyl group transfers as cosubstrate. As much as 12 ATP equivalents are required to regenerate an active SAM (Atkinson, 1977), so 36 ATP equivalents are needed to form one molecule of glycine betaine, which requires three active SAMs. *T. versutus* D301 grows slowly with low biomass, which limits its practical application in biodesulfurization industry (Sharshar et al., 2019). The decreased growth rate of *T. versutus* D301 under high-salt conditions could be attributed to the energy burden caused by the biosynthesis of glycine betaine.

## Conclusion

Glycine betaine was found to be a main compatible solute in *T. versutus* D301, which is widely used as a biocatalyst for desulfurization. Genes associated with nitrogen metabolism of *T. versutus* D301 were significantly upregulated under high-salt conditions, causing the enhanced production of glycine betaine that functions as a main compatible solute to resist osmotic pressure and prevent osmotic lysis. Glycine betaine was synthesized from glycine by TvGMT and TvSDMT in a three-step process of methylation. This work has given us an improved understanding how *Thioalkalivibrio* adapts to extreme environments, which may further contribute to the engineering of *T. versutus* D301 to improve the process of biodesulfurization.

## Data Availability Statement

The datasets presented in this study can be found in online repositories. The names of the repository/repositories and accession number(s) can be found below: National Center for Biotechnology Information (NCBI) BioProject database under accession number PRJNA812740.

## Author Contributions

ML and YZ designed the research. ML, HL, FM, NY, DZ, and GA performed the research and analyzed the data. ML wrote the manuscript under the guidance of YZ. DZ, GA, and HX participated in discussion and revision. All authors have read and approved the final manuscript.

## Conflict of Interest

The authors declare that the research was conducted in the absence of any commercial or financial relationships that could be construed as a potential conflict of interest.

## Publisher’s Note

All claims expressed in this article are solely those of the authors and do not necessarily represent those of their affiliated organizations, or those of the publisher, the editors and the reviewers. Any product that may be evaluated in this article, or claim that may be made by its manufacturer, is not guaranteed or endorsed by the publisher.

## Footnotes

1
https://www.ncbi.nlm.nih.gov/
